# Mirikizumab is associated with rapid and sustained improvements in novel measures of bowel urgency in moderately-to-severely active ulcerative colitis: 28-week results from the LUCENT-URGE trial

**DOI:** 10.1093/ecco-jcc/jjag049

**Published:** 2026-05-11

**Authors:** Silvio Danese, Axel Dignass, David Laharie, Jimmy K Limdi, Radoslaw Kempinski, James D Lewis, Ziad Younes, Erica Cohen, Karla Alaka, William J Eastman, Tian Tian, Isabel Redondo, David T Rubin, Marla Dubinsky

**Affiliations:** IRCCS Ospedale San Raffaele and University Vita-Salute San Raffaele, Milan, Italy; Agaplesion Markus Hospital, Goethe University, Frankfurt/Main, Germany; CHU de Bordeaux, Centre Medico-Chirurgical Magellan, Hôpital Haut-Lévêque, Gastroenterology Department, Université de Bordeaux, INSERM CIC 1401, Bordeaux, France; Northern Care Alliance NHS Foundation Trust and University of Manchester, Manchester, United Kingdom; Wrocław Medical University, Wrocław, Poland; University of Pennsylvania, Philadelphia, PA, United States; Gastroenterology Center of the MidSouth PC, Germantown, TN, United States; Capital Digestive Care, Chevy Chase, MD, United States; Eli Lilly and Company, Indianapolis, IN, United States; Eli Lilly and Company, Indianapolis, IN, United States; Eli Lilly and Company, Indianapolis, IN, United States; Eli Lilly and Company, Indianapolis, IN, United States; University of Chicago Medicine Inflammatory Bowel Disease Center, Chicago, IL, United States; Icahn School of Medicine at Mount Sinai, Icahn School of Medicine, New York, NY, United States

**Keywords:** mirikizumab, IL-23, ulcerative colitis, bowel urgency

## Abstract

**Introduction:**

LUCENT-URGE (NCT05767021) was a Phase 3b, multicenter, open-label, single-arm study investigating bowel urgency (BU) in patients with moderately-to-severely active ulcerative colitis (UC) and BU at baseline, treated with mirikizumab.

**Methods:**

Patients without prior mirikizumab exposure received intravenous mirikizumab 300 mg at weeks (W)0, 4, and 8, followed by subcutaneous mirikizumab 200 mg at W12, 16, 20, and 24. The primary objective was improvement in the validated BU severity measure (Urgency Numeric Rating Scale [UNRS]) at W12. Secondary objectives included improvement in BU at W28, novel measures of stool deferral time (SDT), bowel urgency frequency (BUF), and associations between BU measures. UNRS, BUF, and SDT were collected using a daily diary; the shortest weekly SDT was used for analysis. Baseline observation carried forward was used as the response for the corresponding visit for all missing observations. Missing continuous data were treated as having no change from baseline.

**Results:**

All three BU measures at W12 were sustained or improved through W28. With mirikizumab treatment, 52.2% improved BU severity, 55.1% improved BUF, and 40.7% improved shortest weekly SDT. Patients with shortest weekly SDT ≥ 15 min or no urgency increased from 4.1% at baseline to 29.7% at W28. At W12, 36 (20.9%) achieved clinical remission and 54 (31.4%) achieved endoscopic remission, improving to 62 (36.1%) and 76 (44.2%) at W28, respectively. The safety profile was generally consistent with the known profile.

**Conclusion:**

In the first comprehensive approach assessing complex BU symptoms in UC, mirikizumab was associated with improvements in several BU and clinically related measures through W28.

**Clinical trials registration:**

ClinicalTrials.gov, NCT05767021.

## 1. Introduction

Ulcerative colitis (UC) is a chronic condition marked by inflammation, ulceration, and bleeding in the colon and rectum. It has a relapsing–remitting course, with intermittent flares alternating with periods of remission.^1^ Management paradigms emphasize treating beyond symptoms and to a target that involves the composite assessment of clinical symptoms, patient-relevant outcomes, and endoscopic remission.

Bowel urgency (BU), the sudden and immediate need to have a bowel movement, is a burdensome symptom associated with substantial impacts on quality of life (QoL).^2–4^ More than 80% of patients with UC experience BU, and 50% experience BU at least once a day.^5^ BU is a symptom that can be assessed based on how severe it is experienced by the patient, the frequency it is experienced, and the length of time during which it is possible for the patient to defer defecation after the desire to evacuate.^5^ An estimated 28%-54% of individuals with UC report having a deferral time of 5 min or less, which is substantially shorter compared with patients without UC and urgency.^5^^,^^6^ In a quantitative cross-sectional survey of patients with moderately-to-severely active UC, 45.0% of US and 37.0% of European patients reported wearing diapers, pads, or protection at least once a week in the last 3 months due to the anticipation or fear of fecal urge incontinence.^7^ Although BU has considerable impact on the QoL of patients with UC, data are limited on the impact of treatment on this symptom.

Mirikizumab is an anti-IL-23p19 monoclonal antibody approved for the treatment of UC and Crohn’s disease, which has previously demonstrated long-term clinical efficacy and safety in improving clinical outcomes in patients with UC in the LUCENT-1, -2, and -3 trials.^8^^,^^9^ A greater proportion of patients receiving mirikizumab experienced BU improvement compared with patients receiving placebo, and BU improvement was associated with better clinical outcomes.^10^ Herein, we report the results of the LUCENT-URGE trial, which assessed several novel patient-centric measures of BU improvement, such as bowel urgency frequency and stool deferral time for the first time in UC.

## 2. Methods

### 2.1. Study design

The LUCENT-URGE trial (NCT05767021) was a Phase 3b, open-label, single-arm, 28-week study designed to investigate the relationship of BU with other outcome measures in patients with moderately-to-severely active UC treated with mirikizumab. The study design consists of four periods: a 28-day screening period (period 1), a 12-week treatment period with intravenous mirikizumab (period 2), a 16-week treatment period with subcutaneous mirikizumab (period 3), and a post-treatment follow-up period (period 4). Participants received 300 mg mirikizumab intravenously at Weeks 0, 4, and 8, followed by 200 mg subcutaneously at Weeks 12, 16, 20, and 24 (Figure S1). For periods 1 and 2, participants who entered the study on corticosteroid therapy were required to be on a stable prescribed dose for at least 2 weeks prior to the screening endoscopy and the prescribed dose should be maintained without change throughout these study periods. For participants who entered period 3 on corticosteroid therapy, corticosteroid tapering was initiated at Visit 6 (Week 12). Eligible patients could opt-in to participate in a continued access period. The primary objective was to assess BU severity using the validated Urgency Numeric Rating Scale (UNRS) at Week 12.^11^

Key secondary objectives were the improvement in BU severity and changes in the UNRS score at Week 28; improvements in the novel measures of BU frequency (BUF) and stool deferral time (SDT) at Weeks 12 and 28; and the proportion of participants achieving meaningful improvements in BU severity, BUF, and SDT at these timepoints. Other secondary objectives included the proportion of patients achieving clinical remission and a UNRS score of ≤1; proportion of patients achieving clinical response and a UNRS improvement of ≥3 points from baseline at Weeks 12 and 28; the association between BU severity, BUF, and SDT; and the association between BU severity, BUF, and SDT with UC symptom measures at Weeks 12 and 28 (Table S1).

### 2.2. Patients

Patients aged 18-80 years old were included if they had an established UC diagnosis for at least 3 months; had moderately-to-severely active UC as defined by a Modified Mayo Score (MMS) of 4-9 with a centrally read Mayo endoscopic subscore (ES) of ≥2; had current bowel urgency (UNRS ≥ 3) during screening; and demonstrated an inadequate response, loss of response, or intolerance to conventional or biologic/Janus kinase (JAK) inhibitor/sphingosine-1-phosphate (S1P) receptor modulator therapy. Patients receiving systemic corticosteroids at baseline were required to be on a stable dose for at least 2 weeks prior to the screening endoscopy, with the following upper limits: prednisone ≤20 mg/day (or equivalent), budesonide extended-release ≤9 mg/day (budesonide MMX), or beclomethasone dipropionate 5 mg/day. Patients were excluded if they had Crohn’s disease, unclassified inflammatory bowel disease (IBD), or proctitis only; extensive colonic surgery or other colonic surgery within 6 months; toxic megacolon, intra-abdominal abscess, or stricture/stenosis; history or current evidence of cancer of the gastrointestinal tract or had dysplasia from current surveillance colonoscopy; active tuberculosis, hepatitis C virus, hepatitis B virus, or human immunodeficiency virus. Additional exclusion criteria were past use of anti-IL-23p19 antibodies for any indication; discontinuation of ustekinumab due to loss of response, inadequate response, or intolerance (with exception of discontinuations for other reasons); and failure of more than three approved biologic/JAK/S1P therapies for UC.

### 2.3. Outcome assessments

#### 2.3.1. BU severity (UNRS)

UNRS measured BU from 0 (no urgency) to 10 (worst possible urgency) over the past 24 h. Weekly average scores were calculated as the mean score over a 7-day period, and higher scores indicated worse BU severity.^5^^,^^11^ UNRS clinically meaningful improvement (CMI) is defined as a decrease from the baseline UNRS score* ≥ *3.^10^ UNRS remission is defined as rounded UNRS ≤ 1.

#### 2.3.2. Bowel urgency frequency (BUF)

BUF is a novel single-item patient-reported outcome (PRO) of the number of urgent bowel movements reported in the past 24 h. Response is an integer corresponding to the number of urgent bowel movements reported in the past 24 h.

#### 2.3.3. Stool deferral time (SDT)

SDT is a novel single-item PRO that measured the average duration a participant could delay a bowel movement over the past 24 h. Participants report an integer value representing the average number of minutes they could wait before having a bowel movement or a “no urgency” response during this period. SDT is classified into intervals as: <1 min; 1 to <2 min; 2 to <5 min; 5 to <15 min; ≥15 min (considered a normal SDT in patients in remission with UC) or no urgency.^12^ The shortest weekly SDT is defined as the minimum number of minutes reported over the past 7 days, with “no urgency” responses treated as the largest value. The shortest weekly SDT (minimum value) was selected as the primary summary measure rather than mean or median for both clinical and statistical reasons. Clinically, patients’ daily functioning is often constrained by their worst days; a single episode of very short deferral time can prevent patients from leaving home or participating in daily activities. Statistically, outliers were observed in SDT reporting, suggesting that some patients may have reported deferral time in incorrect units; the minimum function is robust to such extreme values, whereas the mean would be disproportionately affected.

#### 2.3.4. Other relevant outcomes

Clinical remission is defined as: stool frequency (SF) subscore of 0, or SF of 1 with a ≥1-point decrease from baseline; rectal bleeding (RB) subscore of 0; and ES of 0 or 1, excluding friability. Clinical response is defined as a change in the MMS of ≤−2 points and ≤−30% change from baseline, and a change of ≤−1 point in the RB subscore from baseline or an RB score of 0 or 1.^8^  Symptomatic remission is defined as an SF subscore = 0, or SF = 1 with a ≥1-point decrease from baseline, and an RB subscore = 0.^10^ Endoscopic normalization is defined as an ES of 0.^13^ Endoscopic remission is defined as an ES of 0 or 1, excluding friability.^8^

The Inflammatory Bowel Disease Questionnaire (IBDQ) is a 32-item PRO that measured four aspects of participants’ lives: bowel symptoms, systemic symptoms, emotional function, and social function. Response options are on a seven-point Likert scale in which 7 denotes “not a problem at all” and 1 denotes “a very severe problem,” with a higher score indicating a better QoL.^14^

The Patient Global Rating of Severity (PGR-S) is a single-item PRO designed to assess the UC symptom severity in participants over the past 24 h using a six-point scale (1 “none”; 6 “very severe”).^15^ The Patient’s Global Impression of Change (PGI-C) is a single-item PRO designed to assess the participant’s rating of change in their UC symptom severity since they started taking mirikizumab. Responses are graded on a seven-point Likert scale and ranged from 1 “very much better” to 7 “very much worse.”^15^

Absorbent Product Use for Bowel Urgency is a single-item PRO of the participant’s feeling of need to wear an adult diaper, pad, or protection because of bowel urgency over the past 2 months. Responses range from “never” (0 times) to “always” (at least once a day).

The Wexner Incontinence Score is a five-item PRO designed to assess the participant’s frequency and type of incontinence (solid stool, liquid stool, gas, pads, or lifestyle altered) over the past 2 months. Responses ranged from “never” (0 times) to “always” (at least once a day).^16^

Other PROs included Fatigue NRS, Abdominal Pain NRS, and Nocturnal Stool. Fatigue NRS and Abdominal Pain NRS are single patient-reported items measured on an 11-point scale ranging from 0 (no fatigue/no pain) to 10 (fatigue as bad as you can imagine/worst possible pain) in the past 24 h. Nocturnal Stool is a single-item instrument used to record the number of stools patients had during the night (or day, for shift workers) causing them to wake from sleep.^17^ Outcome measures are defined in Table S1.

### 2.4. Statistical analysis

The sample size was determined by considering the precision of the estimated correlation between endpoints, and a proposed sample size of 160 participants was considered sufficient to have >90% power to detect a significant change from baseline in UNRS at Week 12. Descriptive summaries with 95% confidence interval (CI) for the average change from baseline at Week 12 and Week 28 were provided for continuous measures. If more than 3 of the 7 days were missing, the average score and change from baseline were marked as missing. Participants who discontinued early were considered to have no improvement from baseline. Categorical efficacy endpoints were summarized using frequency counts and proportions.

Missing data from primary and clinically meaningful improvement secondary endpoints were considered as no change from baseline for early discontinuations. Non-responder imputation (NRI) was used as the primary imputation method for missing binary data, and baseline observation carried forward (BOCF) was used for continuous data. These approaches align with a composite estimand applied to treatment discontinuation, targeting outcomes in all randomized patients regardless of treatment adherence, whereby treatment discontinuation is incorporated into the endpoint rather than missing. Correlation values were derived from observed data, excluding missing data at the specified time point. Spearman correlation coefficient, a rank-based method known for its robustness to skewed or non-normally distributed data, was used to assess the strength and direction of the pairwise relationships between measures. Summaries of binary and continuous endpoints for correlation analyses were included as observed estimates, not regarded as missing data imputations. For binary endpoints, 95% CIs were calculated using the asymptotic method (normal approximation to the binomial distribution) without continuity correction. For continuous endpoints, 95% CIs were calculated using a normal approximation. For Spearman correlation coefficients, 95% CIs were derived using Fisher’s z transformation. No adjustment for multiple comparisons was performed.

### 2.5. Ethical considerations

The study was conducted in accordance with consensus ethics principles derived from international ethics guidelines, including the Declaration of Helsinki and Council for International Organizations of Medical Sciences (CIOMS) International Ethical Guidelines, applicable ICH GCP Guidelines, and applicable laws and regulations. All authors had access to the study data and reviewed and approved the final manuscript.

## 3. Results

### 3.1. Patient demographics and characteristics

**Figure 1.**
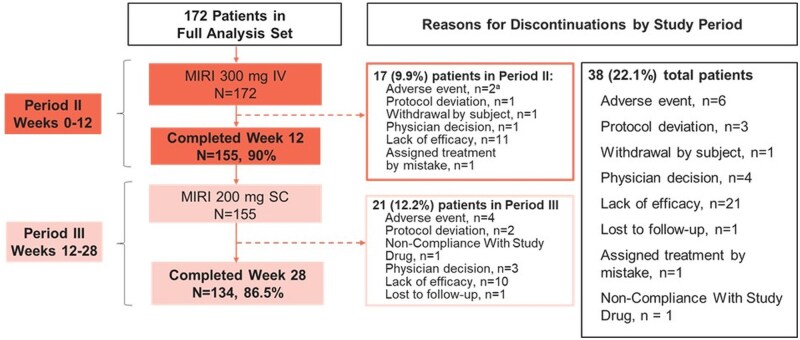
Treatment disposition. Twelve-week intravenous MIRI dosing period—full analysis set (LUCENT-URGE). IV, intravenous; MIRI, mirikizumab; *N*, number of patients in the analysis population; *n*, number of patients in the specified category; SC, subcutaneous. ^a^Colitis ulcerative and large intestinal polyp.

The study enrolled 172 patients across eight countries. Of these patients, 155 (90.1%) completed Week 12, and of these Week 12 completers, 134 (86.5%) completed Week 28 (Figure 1).

The mean age of the patients was 41.1 years, with a majority being male (101, 58.7%) and White (150, 93.2%). The mean disease duration was 8.0 years. Among the participants, 61 (35.5%) had moderately active UC, and 110 (64.0%) had severely active UC. At baseline, 78 (45.3%) patients had extensive UC or pancolitis. Baseline mean BU severity was 6.9, and BUF episodes were 6.9 times/day. Additionally, 51.7% of the patients had concomitant corticosteroid use at baseline (prednisone equivalent mean dose 15.5 mg), and 36.6% had prior failure with one or more advanced therapies (Table 1).

**Table 1.** Baseline demographics.

	MIRI 300 mg IV/200 mg SC *N* = 172
**Age (years), mean (SD)^a^**	41.1 (14.4)
**Male, *n* (%)**	101 (58.7)
**BMI (kg/m^2^), mean (SD)^b^**	25.9 (5.9)
**Race, *n* (%)^c^**	
**White**	150 (93.2)
**Black or African American**	5 (3.1)
**Asian**	4 (2.5)
**Other^d^**	2 (1.2)
**Geographic region, *n* (%)**	
**North America**	33 (19.2)
**Europe**	139 (80.8)
**Duration of ulcerative colitis (years), mean (SD)^e^**	8.0 (8.2)
**Disease location, *n* (%)**	
**Ulcerative proctitis**	4 (2.3)
**Left-sided UC (distal UC)**	90 (52.3)
**Extensive ulcerative colitis/pancolitis**	78 (45.3)
**Modified Mayo score category, *n* (%)**	
**Moderate (4-6)**	61 (35.5)
**Severe (7-9)**	110 (64.0)
**UNRS score [range 0-10], mean (SD)**	6.9 (1.6)
**SDT, min, *n* (%)**	
**<1 min**	34 (19.8)
**≥1 to <2 min**	59 (34.3)
**≥2 to <5 min**	57 (33.1)
**≥5 to <15 min**	15 (8.7)
**≥15 min or no urgency**	7 (4.1)
**BUF, times/day**	6.9 (3.7)
**Stool frequency, Mayo subscore [range 0-3], mean (SD)**	2.6 (0.7)
**Rectal bleeding, Mayo subscore [range 0-3], mean (SD)**	1.6 (0.8)
**Nocturnal stool, *n* [number reported], mean (SD)**	1.9 (1.8)
**Loose stool [range 6-7], *n* (%)**	156 (90.7)
**Abdominal pain NRS score [range 0-10], mean (SD)**	5.7 (2.4)
**Fatigue NRS score [range 0-10], mean (SD)**	6.4 (2.3)
**PGR-S score [range 1-6], mean (SD)**	4.6 (0.6)
**IBDQ total score [range 32-224], mean (SD)**	106.2 (27.9)
**Wexner incontinence score [range 0-4], mean (SD)**	10.9 (4.8)
**Solid stool**	1.4 (1.4)
**Liquid stool**	2.6 (1.3)
**Gas**	2.9 (1.3)
**Pads**	1.2 (1.6)
**Lifestyle altered**	2.9 (1.3)
**Absorbent product use for BU [range 0-4], mean (SD)**	1.4 (1.6)
**Prior corticosteroid failure, *n* (%)^f^ ^,^** ^g^	4 (2.3)
**Prior biologic failure, *n* (%)^h^ ^,^** ^g^	63 (36.6)
**Prior S1P failure, *n* (%)^i^ ^,^** ^g^	2 (1.2)
**Prior JAK inhibitor failure, *n* (%)^j^ ^,^** ^g^	23 (13.4)

Baseline is defined as the last non-missing value prior to the first dose of study intervention.

a Age in years is calculated as length of the time interval from the imputed date of birth (July 1 in the year of birth collected in the eCRF) to the informed consent date.

b Body mass index (BMI) is calculated as: BMI (kg/m^2^) = weight (kg)/(height (m))^2^.

c Number of participants who reported race was 161/172; data were missing for 11 participants.

d One (0.6%) American Indian/Alaska Native and one (0.6%) Multiple.

e Length of the interval from the date of UC diagnosis to the date of informed consent.

f Options on the prior therapy eCRF for corticosteroids include prednisone, prednisolone, cortiment MMX, beclomethasone, budesonide, methylprednisone, and hydrocortisone.

g Failure defined as reasons for prior treatment discontinuation are inadequate response, loss of response, intolerance to medication.

h Options on the prior therapy eCRF for biologic agents include infliximab, infliximab biosimilar, adalimumab, adalimumab biosimilar, alemtuzumab, brazikumab, certolizumab, guselkumab, interferon therapy, natalizumab, risankizumab, rituximab, tildrakizumab, ustekinumab, vedolizumab, visilizumab.

i Options for prior therapy eCRF for S1P include: etrasimod, ozanimod.

j Options on the prior therapy eCRF for JAK inhibitor include: tofacitinib, filgotinib, upadacitinib.

Abbreviations: BMI, body mass index; BU, bowel urgency; BUF, bowel urgency frequency; IBDQ, Inflammatory Bowel Disease Questionnaire; IV, intravenous; JAK, Janus kinase; MIRI, mirikizumab; *N*, number of patients in the analysis population; *n*, number of patients in the specified category; NRS, Numeric Rating Scale; PGR-S, Patient’s Global Rating of Severity; SC, subcutaneous; SD, standard deviation; SDT, shortest weekly stool deferral time; S1P, sphingosine-1-phosphate; UNRS, Urgency Numeric Rating Scale.

### 3.2. Improvements in BU measures

**Figure 2.**
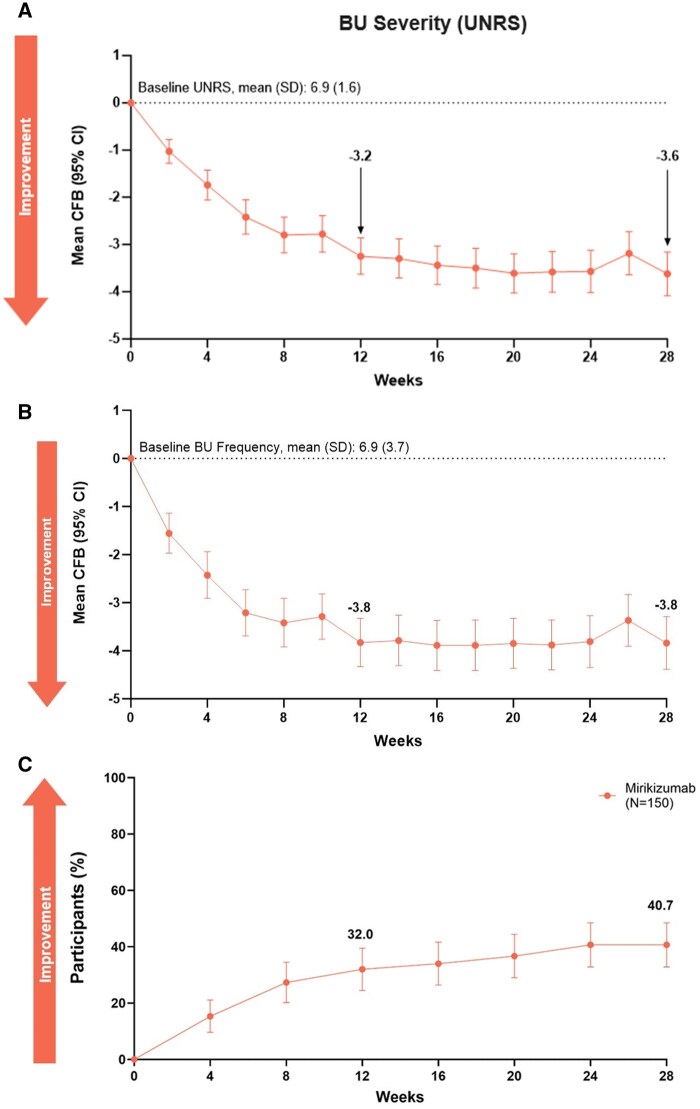
BU severity (A), BU frequency (B), and SDT (C) improvement by week 28. BOCF, baseline observation carried forward; BU, bowel urgency; CFB, change from baseline; CI, confidence interval; IV, intravenous; *N*, number of patients in the analysis population; NRI, non-responder imputation; NRS, Numeric Rating Scale; SC, subcutaneous; SDT, shortest weekly stool deferral time. (A) Urgency NRS (BU severity), BOCF. (B) BU frequency, BOCF. (C) Proportion of patients with SDT ≥ 5 min or no urgency in patients with baseline SDT < 5 min, NRI.

Improvements were observed across all BU measures through 28 weeks of continuous treatment. The primary endpoint result of BU severity change from baseline at Week 12 showed a decrease in mean UNRS score from 6.9 at baseline to 3.7 at Week 12, and a further reduction to 3.3 at Week 28 (Figure 2A). Mean BUF decreased from 6.9 times/day at baseline to 3.1 times/day at Week 12, which was sustained to Week 28 (Figure 2B). Clinically relevant improvement in shortest weekly SDT was observed from baseline through Week 28 (Figure S2, Video S1). Among patients with baseline shortest weekly SDT < 5 min, 48 (32.0%) had shortest weekly SDT of ≥5 min or no urgency at Week 12, increasing to 61 (40.7%) at Week 28 (Figure 2C). At Week 12, 92 (53.5%) patients achieved UNRS CMI, and 100 (58.1%) patients achieved UNRS CMI at Week 28 (Figure S3). UNRS remission was achieved by 38 (22.1%) patients at Week 12, and 65 (37.8%) patients at Week 28.

### 3.3. Improvements in clinical outcome response rates

At Week 12, 108 (62.8%) patients achieved clinical response and 36 (20.9%) achieved clinical remission. By Week 28, 106 (61.6%) patients achieved clinical response and 62 (36.1%) achieved clinical remission. Of these 62 clinical remitters at Week 28, 39 (62.9%) achieved corticosteroid-free clinical remission. At Week 12, 54 (31.0%) patients achieved endoscopic remission, and 76 (44%) achieved endoscopic remission at Week 28, while endoscopic normalization was achieved by 13 (7.6%) patients at Week 12 and by 35 (20%) at Week 28. Symptomatic remission, defined as an SF subscore = 0, or SF = 1 with a >1-point decrease from baseline, and an RB subscore = 0, also improved in 77 (45%) patients at Week 12 and 94 (55%) at Week 28 (Table S2).

At Week 12, 76 (44.2%) patients achieved both clinical response and BU CMI, and 86 (50.0%) patients achieved both clinical response and BU CMI at Week 28. For clinical remission and BU CMI, 24 (14.0%) and 56 (32.6%) patients achieved both endpoints at Weeks 12 and 28, respectively. At Week 12, nine (5.2%) patients achieved both clinical remission and BU remission (rounded UNRS score of ≤1), and 36 (20.9%) patients achieved both endpoints at Week 28. Baseline characteristics between patients who achieved both clinical remission and BU remission versus those who achieved clinical remission but not BU remission at Week 28 are shown in Table S3. At Week 28, 16 (45.7%) patients with ES = 0 achieved BU remission (UNRS ≤ 1) and ES = 0; 23 (56.1%) patients with ES = 1 achieved BU remission and ES = 1 (Table S4).

### 3.4. Change in UC symptoms measures

Improvements in UC symptoms observed from baseline to Week 12 were maintained through Week 28 (Table 2). At baseline, patients had a mean absolute stool frequency of 8.4, which decreased to 4.3 at Week 12 and to 4.4 at Week 28. Rectal bleeding improved from a mean score of 1.6 at baseline to 0.4 at Week 12 and to 0.5 at Week 28. The mean number of nocturnal stools decreased from 1.9 at baseline to 0.6 at Week 12 and to 0.8 at Week 28. Mean stool consistency score improved from 6.4 at baseline on the Bristol Stool Scale to 5.4 at Weeks 12 and 28. Abdominal pain severity decreased from a mean score of 5.7 at baseline to 3.0 at Week 12 and to 2.8 at Week 28. Fatigue improved from a mean score of 6.4 at baseline to 4.3 at Week 12 and to 4.1 at Week 28. Mean PGR-S score improved from 4.6 at baseline to 3.2 at Week 12 and to 3.0 at Week 28. In the PGI-C, patients reported their UC symptoms as “very much better” or “much better” at both Weeks 12 and 28. Mean total IBDQ score increased from 106 at baseline to 157 at Week 12 and further to 163 at Week 28. Baseline mean absorbent product use score was 1.4, indicating moderate use of absorbent products. By Week 12, the absorbent product use score decreased to 0.9, with further improvements observed at Week 28. The Wexner Incontinence Score for subscores of solid stool, liquid stool, gas, pads, and lifestyle altered improved to “rarely” from baseline to Week 28, with liquid stool and lifestyle altered showing the greatest change.

**Table 2.** Change in PRO measures from baseline at week 12 and week 28.

	Baseline	Week 12	Week 28	Week 12 CFB	Week 28 CFB
**PRO, mean (SD) (95% CI)**					
**Absolute SF, *n* [number reported]**	8.4 (4.2)	4.3 (2.9)	4.4 (3.7)	−4.0 (3.7)	−4.0 (4.1)
		(3.9, 4.8)	(3.8, 4.9)	(−4.6, −3.5)	(−4.6, −3.4)
**SF Mayo Subscore [range 0-3]**	2.6 (0.7)	1.4 (1.0)	1.3 (1.1)	−1.2 (1.0)	−1.3 (1.1)
		(1.2, 1.5)	(1.1, 1.5)	(−1.4, −1.1)	(−1.5, −1.1)
**Rectal bleeding, Mayo subscore [range 0-3]**	1.6 (0.8)	0.4 (0.8)	0.5 (0.8)	−1.1 (0.9)	−1.1 (1.0)
		(0.3, 0.6)	(0.3, 0.6)	(−1.3, −1.0)	(−1.2, −0.9)
**Nocturnal stools, *n* [number reported]**	1.9 (1.8)	0.6 (1.1)	0.8 (1.6)	−1.3 (1.4)	−1.1 (1.3)
	(1.6, 2.2)	(0.5, 0.8)	(0.5, 1.0)	(−1.5, −1.1)	(−1.3, −0.9)
**Stool consistency score [range 1-7]**	6.4 (0.7)	5.4 (1.2)	5.4 (1.3)	−1.0 (1.3)	−1.0 (1.4)
	(6.3, 6.5)	(5.2, 5.6)	(5.2, 5.6)	(−1.2, −0.8)	(−1.2, −0.8)
**Abdominal pain NRS score [range 0-10]**	5.7 (2.4)	3.0 (2.5)	2.8 (2.8)	−2.7 (2.6)	−2.9 (2.8)
	(5.3, 6.1)	(2.6, 3.4)	(2.3, 3.2)	(−3.1, −2.3)	(−3.4, −2.5)
**Fatigue NRS score [range 0-10]**	6.4 (2.3)	4.3 (2.8)	4.1 (3.1)	−2.1 (2.6)	−2.3 (2.9)
	(6.0, 6.7)	(3.9, 4.7)	(3.6, 4.5)	(−2.5, −1.7)	(−2.7, −1.9)
**PGR-S score [range 1-6]**	4.6 (0.6)	3.2 (1.2)	3.0 (1.4)	−1.4 (1.2)	−1.6 (1.4)
	(4.5, 4.7)	(3.0, 3.4)	(2.8, 3.2)	(−1.5, −1.2)	(−1.8, −1.4)
**PGI-C score (no baseline) [range 1-7]**	N/A	2.0 (1.0)	1.7 (1.0)	N/A	N/A
**IBDQ total score [range 32-224]**	106 (27.9)	157 (42.6)	163 (44.6)	51 (37.5)	56 (45.8)
		(150.8, 163.8)	(156.4, 170.1)	(45.4, 57.1)	(49.2, 63.5)
**Wexner incontinence scale [range 0-4]**					
**Solid stool**	1.4 (1.4)	1.1 (1.3)	0.9 (1.3)	−0.2 (1.5)	−0.5 (1.4)
**Liquid stool**	2.6 (1.3)	1.4 (1.4)	1.3 (1.5)	−1.2 (1.6)	−1.3 (1.6)
**Gas**	2.9 (1.3)	2.2 (1.5)	1.9 (1.5)	−0.6 (1.5)	−0.9 (1.5)
**Pads**	1.2 (1.6)	0.8 (1.4)	0.6 (1.3)	−0.5 (1.4)	−0.6 (1.4)
**Lifestyle altered**	2.9 (1.3)	1.6 (1.5)	1.3 (1.5)	−1.3 (1.5)	−1.6 (1.6)
**Absorbent product use for BU [range 0-4]**	1.4 (1.6)	0.9 (1.4)	0.7 (1.3)	−0.6 (1.3)	−0.7 (1.3)
		(0.6, 1.1)	(0.5, 0.9)	(−0.8, −0.4)	(−0.9, −0.5)

Abbreviations: BU, bowel urgency; CFB, change from baseline; CI: confidence interval; IBDQ, Inflammatory Bowel Disease Questionnaire; MIRI, mirikizumab; *n*, number of patients in the specified category; N/A, not available; NRS, Numeric Rating Scale; PGI-C, Patient’s Global Impression of Change; PGR-S, Patient’s Global Rating of Severity; PRO, patient-reported outcome; SD, standard deviation; SF, stool frequency.

### 3.5. Correlations

**Figure 3.**
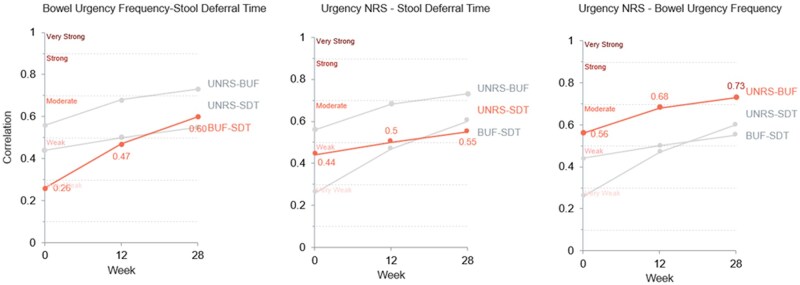
Correlations between BU measures at weeks 12 and 28: Urgency NRS (BU severity), BU frequency, and stool deferral time. Absolute value of Spearman correlation coefficients between the three measures. BU, bowel urgency; BUF, bowel urgency frequency; BUS, bowel urgency severity; CFB, change from baseline; NRS, Numeric Rating Scale; SDT, shortest weekly stool deferral time.

Spearman correlation coefficients for BU severity and BUF at Weeks 12 and 28 indicate moderate-to-strong correlations throughout the study. At Week 12, the correlation coefficient (95% CI) between BU severity and frequency was 0.68 (0.59, 0.76), and at Week 28, it was 0.73 (0.64, 0.80). Shortest weekly SDT had moderate correlations with BU severity at Week 12, and moderate correlations with both BU severity and frequency at Week 28 (Figure 3). BU severity and BUF showed directionally positive Spearman correlations; shortest weekly SDT was inversely correlated with other measures, such that an increase in shortest weekly SDT minutes corresponded with a reduction or improvement in those measures.

BU severity, BUF, and shortest weekly SDT measures overall showed stronger correlations with other patient-reported UC symptom measures at Weeks 12 and 28 compared with baseline (Table 3). The strongest correlations were observed between BU severity and BUF with stool frequency, abdominal pain, and fatigue. The three BU measures were less associated with rectal bleeding. Stool frequency showed moderate correlations with BU severity and BUF consistently, and Fatigue NRS had a moderate correlation with BU severity. PGR-S had moderate correlations with all 3 BU measures at Week 28.

**Table 3.** Correlations between bowel urgency and UC symptom measures.

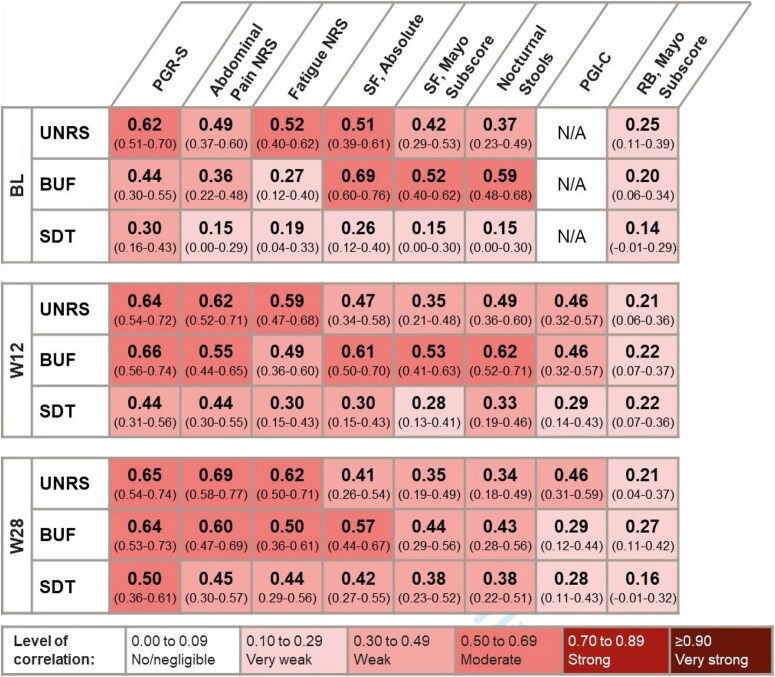

Absolute values of Spearman correlation coefficients (95% CI).

Abbreviations: BUF, bowel urgency frequency; CI, confidence interval; *n*, number of patients in the specified category; NRS, Numeric Rating Scale; PGI-C, Patient’s Global Impression of Change; PGR-S, Patient’s Global Rating of Severity; RB, rectal bleeding; SDT, stool deferral time; SF, stool frequency.

### 3.6. Safety

Of the 172 patients who received mirikizumab, 107 (62.2%) experienced at least one treatment-emergent adverse event (TEAE; Table 4). Most of these adverse events were mild to moderate in severity, and 8 (4.7%) patients had severe TEAEs. Serious adverse events were reported in nine (5.2%) patients. There were no deaths reported, and eight (4.7%) patients discontinued treatment due to adverse events. No malignancies, suicide/self-injury, or major adverse cardiovascular events were reported. Serious infections were reported for two (1.2%) patients (one joint abscess and one pneumonia). Opportunistic infection was reported in one (0.6%) patient with herpes zoster. Adjudicated cerebrocardiovascular events were reported in three (1.7%) patients with a prior history of the event and/or known risk factors, with two deep vein thrombosis and one pulmonary embolism. None of the adjudicated events were assessed as associated with mirikizumab treatment, and no patients were discontinued due to these events.

**Table 4.** Overview of adverse events and AEs of particular interest.

	**MIRI 300** **mg IV/200** **mg SC *N*** = **172**
**Patients with ≥1 TEAE, *n* (%)**	107 (62.2)
**TEAE by severity^a^**	
**Mild**	55 (32.0)
**Moderate**	44 (25.6)
**Severe**	8 (4.7)
**Discontinuation from study treatment due to adverse event (including death)**	8 (4.7)
**Death**	0
**Serious adverse event**	9 (5.2)
**Colitis ulcerative**	2 (1.2)
**Deep vein thrombosis**	2 (1.2)
**Joint abscess**	1 (0.6)
**Pulmonary embolism**	1 (0.6)
**Anemia**	1 (0.6)
**Pneumonia**	1 (0.6)
**Clavicle fracture**	1 (0.6)
**AEs of particular interest**	
**Infection, *n* (%)**	39 (22.7)
**Serious infection**	2 (1.2)
**Opportunistic infection (narrow)**	1 (0.6)
**Immediate hypersensitivity reactions (narrow), *n* (%)**	3 (1.7)
**Infusion-site reactions (high-level term), *n* (%)**	3 (1.7)
**Injection-site reactions (high-level term), *n* (%)**	5 (2.9)
**CCV event (adjudicated and confirmed), *n* (%)**	3 (1.7)
**MACE**	0
**Malignancy, *n* (%)**	0
**Depression excluding suicide/self-injury (narrow), *n* (%)**	1 (0.6)
**Suicide/self-injury (narrow), *n* (%)**	0
**Hepatic event (narrow), *n* (%)**	7 (4.1)

a Patients with multiple TEAE occurrences of the same event are counted under the highest severity.

Serious infection: 1 joint abscess and 1 pneumonia; OI: 1 herpes zoster; CCV: 2 for deep vein thrombosis and 1 for pulmonary embolism.

MedDRA version 27.1.

Abbreviations: %, percentage based on *N*; CCV, cerebrocardiovascular; IV, intravenous; MACE, major adverse cardiovascular event; MIRI, mirikizumab; *N*, number of patients in the analysis set; *n*, number of patients in the specified category; SC, subcutaneous; TEAE, treatment emergent adverse event.

## 4. Discussion

In this multicenter, Phase 3b, open-label, single-arm trial of patients with moderately-to-severely active UC and BU, we observed that a high proportion achieved improvement in three BU measures through 28 weeks of treatment (52.2% BU severity; 55.1% BUF; 40.7% shortest weekly SDT) with mirikizumab. Clinical endpoints also generally showed marked improvement through Week 28. Of the 62 patients achieving clinical remission, 90.3% had BU CMI and 58.1% had BU remission. Substantial improvements in patient-reported fecal incontinence score, a clinically important measure affecting patient QoL, were also observed over 28 weeks. Most correlations between BU measures and other symptoms remained stable or improved during the study. Moderate correlations between all three BU measures and PGR-S support that BU is an important component of patients’ overall symptom burden.

UNRS results showed consistency with previous data, supporting the association between mirikizumab treatment and resolution of BU severity and improvement in QoL.^10^^,^^17^ In this study, 36% of patients achieved clinical remission at Week 28 while 20.9% of patients achieved concomitant clinical and BU remission, indicating almost 50% of patients in clinical remission still have BU. When assessing the association of BU remission with achieving endoscopic normalization (45.7%) versus endoscopic score of 1 (56.1%), there was no sign in favor of achieving a more stringent endpoint. A prior study showed that only achieving both endoscopic normalization and histologic remission (Geboes score ≤ 2B.0) was associated with BU remission (*P* < .05).^13^ These results align with previous observations that a number of patients with clinical remission or endoscopic healing (and thus no rectal bleeding and normal stool frequency) still experienced bowel urgency.^3–6^ At baseline, patients with persistent BU despite clinical remission more frequently reported severe urgency (UNRS > 6: 80.8% vs 69.4%) and a higher prevalence of loose stool (88.5% vs 86.1%). These patients also demonstrated more severe baseline disease activity (severe MMS: 69.2% vs 52.8%) and lower health-related quality of life (IBDQ ≥ 100: 34.6% vs 52.8%). Collectively, these findings underscore that clinical remission alone may be insufficient to reflect the full spectrum of disease burden in UC. While urgency is associated with other clinical outcomes, it is not completely captured using standard assessments and may reflect additional impact on gastrointestinal motor and sensory function.^4^^,^^18^

Current treatment paradigms for UC increasingly emphasize achieving mucosal healing rather than solely focussing on symptomatic remission with the aim of reducing long-term disability and enhancing QoL. Recognizing its high ranking among disruptive symptoms and its association with corticosteroid use, hospitalization, and surgery complicating IBD, BU has been included as a goal in recent clinical trials for UC.^10^^,^^19^^,^^20^ Recent American College of Gastroenterology clinical guidelines highlighted the need to assess urgency during diagnosis and stated urgency resolution as an important treatment goal, ^21^^,^^22^ providing much needed impetus to characterize this endpoint for clinical practice. The selection of moderately-to-severely active patients with UC and bowel urgency to target BU improvement in addition to clinical and endoscopic is a novel strategy that enhances patient-centric comprehensive disease management.

LUCENT-URGE is the first study to enroll only patients with BU at baseline, explore multiple measures of urgency and their correlations, and show a treatment-driven improved deferral time and reduction in BU frequency. The results here showed that in a treat-through design up to 6 months, mirikizumab provides symptomatic, clinical, and endoscopic improvement and remission consistent with LUCENT-1 and LUCENT-2 efficacy data.^8^ These data provide novel observations that improvements in measures of BU are directionally aligned with improvements in clinical response and remission and that patients may continue to have improvement in BU after the first 12 weeks of treatment with mirikizumab.

The inclusion of three measures of BU in the LUCENT-URGE trial—the validated UNRS (BU severity), and the novel assessments of BUF and shortest weekly SDT—provide insights that can be used for the design of future studies. The three BU measures showed correlation coefficients of ≥0.4 at all time points with the exception of BUF with shortest weekly SDT. The correlation of BU severity (UNRS) with BUF was particularly strong, suggesting that it may not be necessary to measure both in future trials. BUF is most dependent on bowel movement frequency and as expected had the strongest correlation of the three measures with the stool frequency measure. The UNRS is the easiest to implement and probably has the greatest statistical efficiency when used as a continuous measure, but it may be the hardest to translate into clinical expectations for patients. In contrast, shortest weekly SDT is not dependent on stool frequency. Shortest weekly SDT had the weakest correlation with stool frequency at Weeks 0 and 12, and a similar correlation with stool frequency to UNRS at Week 28. SDT has the advantage of being easily translatable to patients and is influenced less than UNRS by between-individual differences in rating the severity of BU. As such, it appears that measuring UNRS and shortest weekly SDT in future trials may provide an ideal combination of statistical efficiency and clinically translatable information. If only one measure can be implemented, the UNRS can be utilized as both a continuous and categorical measure to improve its translatability to patients.

The safety profile of LUCENT-URGE was consistent with those of LUCENT-1 and LUCENT-2, with similar frequency of TEAEs (∼60%).^8^ LUCENT-URGE had numerically higher numbers of discontinuations due to adverse events and serious adverse events. Similar frequencies of adverse events of particular interest were reported, with serious infection of 1%, opportunistic infections of less than 1%, and no major adverse cardiovascular event. When considered alongside the observed efficacy, the benefit–risk balance of mirikizumab remained favorable in this symptom-enriched UC population.

The LUCENT-URGE trial was an open-label and single-arm study. This was the first study to enrich a BU population and explore additional aspects of BU beyond severity affecting QoL in patients with moderately-to-severely active UC. A limitation of this study is its single-arm design, which prevents causal inference and comparison of BU outcomes with a placebo or active comparator. Also, there was a lack of published psychometric validation of the novel BU measures. Notably, the results for traditional endpoints measured in UC clinical trials were nearly identical to those of LUCENT-1 and LUCENT-2, demonstrating important reproducibility in this treat-through design trial that may be more representative of the effectiveness expected in clinical practice. Most of the patients were White and from North America and Europe. Thus, the extent that these results are generalizable to other populations remains unknown. Additionally, outliers were observed in the reporting of SDT, suggesting that a few patients may have misunderstood the question and answered their deferral time in days versus in minutes as expected. Given the perceived anomalies in the data, SDT was analyzed with the shortest weekly stool deferral time, which handled extreme values from daily diary records over a 7-day period by calculating the minimum minutes of reported SDT in the past 7 days. The Spearman rank’s correlation coefficient, which is robust to outliers of SDT, was used to assess SDT relationships with other measures, and the correlation directions aligned with expectations.

## 5. Conclusion

In summary, the 28-week results from the LUCENT-URGE trial indicate that mirikizumab treatment was associated with rapid and sustained improvements in BU severity, frequency, and SDT in patients with moderately-to-severely active UC. The safety profile of mirikizumab was consistent with previously reported data. To our knowledge, LUCENT-URGE is the first study in UC to comprehensively evaluate the full spectrum of BU symptoms and measures. Our findings underscore the importance of managing burdensome patient-centric symptoms such as BU in the comprehensive and holistic care of people living with UC.

## Supplementary Material

jjag049_Supplementary_Data

## Data Availability

Lilly provides access to all individual participant data collected during the trial, after anonymization. Data are available to request 6 months after the indication studied has been approved in the US and EU and after primary publication acceptance, whichever is later. No expiration date of data requests is currently set once data are made available. Access is provided after a proposal has been approved by an independent review committee identified for this purpose and after receipt of a signed data sharing agreement. Data and documents, including the study protocol, statistical analysis plan, clinical study report, and blank or annotated case report forms, will be provided in a secure data sharing environment. For details on submitting a request, see the instructions provided at www.vivli.org.
