# Supplementary material for: Mirikizumab is associated with rapid and sustained improvements in novel measures of bowel urgency in moderately-to-severely active ulcerative colitis: 28-week results from the LUCENT-URGE trial
Source: J Crohns Colitis. 2026 May 11;20(5):jjag049. doi: 10.1093/ecco-jcc/jjag049 (PMC13158796; doi:10.1093/ecco-jcc/jjag049)

Supplemental Table 1. Patient Reported Outcomes Daily Measures/Questions

| **Measure** | **Daily Questions** | **Daily Responses** |
| --- | --- | --- |
| **Urgency NRS**  **(BU severity)** | ‘How severe was your urgency (sudden or immediate need) to have a bowel movement in past 24 hours?’ | ___ points on the 11-point NRS  (‘0=No urgency’, … ‘10=Worst possible urgency’) |
| **BU Frequency** | ‘How many times did you experience bowel urgency in the past  24 hours?’ | ‘___ times’ or ‘¨ Did not experience bowel urgency in the past  24 hours (0)’ |
| **SDT** | ‘In the past 24 hours, how many minutes on average could you wait from the time you felt the urge to have a bowel movement before you had to rush to the toilet?’ | ‘___ minutes’ or ‘¨ Did not feel urge to have bowel movement in the past 24 hours (N/A)’ |
| **Stool Frequency** | ‘How many stools did you have in the past 24 hours?’ | ___ (Number) |
| **Nocturnal Stools** | ‘How many stools did you have during the night causing you to waken from sleep?’ | ___ (Number) |
| **Absorbent Product Use for BU** | ‘To manage your bowel urgency over the past two months, how often did you feel the need to wear an adult diaper, pad, or protection because of bowel urgency?’ | 5 options: ‘🞎 Never (0), 🞎 Rarely (<1/month), 🞎 Sometimes (<1/week, ≥1/month), 🞎 Usually (<1/day, ≥1/week), 🞎 Always (≥1/day)’ |
| **Wexner Incontinence Score** | For 5 questions asked about incontinence (solid/liquid stool, gas, pad use, lifestyle altered) indicate frequency over the past 2 months | 5 options: ‘🞎 Never (0), 🞎 Rarely (<1/month), 🞎 Sometimes (<1/week, ≥1/month), 🞎 Usually (<1/day, ≥1/week), 🞎 Always (≥1/day)’ |
| **Rectal Bleeding** | ‘Please select the most severe category that describes amount of blood in your stool in the past 24 hours’ | 4 options: ‘¨ No blood seen (0), ¨ Streaks of blood (1), ¨ Obvious blood (2), ¨ Blood alone (3)’ |
| **Abdominal Pain NRS** | ‘Please rate the severity of your abdominal pain by selecting the number that best describes your worst abdominal pain in the past  24 hours.’ | 11-point NRS (‘0=No pain, … 10=Worst possible pain’) |
| **Fatigue NRS** | ‘Please rate your fatigue (feeling tired or worn out) by selecting the one number that best describes your WORST level of fatigue during the past 24 hours.’ | 11-point NRS (‘0=No fatigue, … 10=As bad as you can imagine’) |
| **PGR-S** | ‘How would you rate your overall ulcerative colitis symptoms over the past 24 hours?’ | 6 options (‘¨ 1=None, … ¨ 6=Very severe’) |
| **PGI-C** | ‘Select the response that best describes how your UC symptoms  are now, compared to how they were before you starting taking  this medication’ | 7 options: (‘¨ 1=Very much better, … ¨ 7=Very much worse’) |
| **IBDQ** | 32-item questionnaire designed to measure effects of IBD on daily function and QoL, over the past 2 weeks | 7 options/wording varies yet generally indicates: ‘¨ 1 (All of the time),  ¨ 2 (Most of the time), ¨ 3 (A good bit of the time),  ¨ 4 (Some of the time), ¨5 (A little of the time),  ¨ 6 (Hardly any of the time), ¨ 7 (None of the time)’ |

BU=Bowel Urgency; IBDQ=Inflammatory Bowel Disease Questionnaire; n=Number of Responses in the Specified Category; N/A=Not Applicable; NRS=Numeric Rating Scale; PGI-C=Patient’s Global Impression of Change; PGR-S=Patient’s Global Rating of Severity; PRO=Patient-reported Outcome; QoL=Quality of Life; SDT=Stool Deferral Time; UC=Ulcerative Colitis.

Supplemental Table 2. Clinical Outcome Response Rates at Week 12 and Week 28

|  | LUCENT-1 | LUCENT-URGE | LUCENT-URGE |
| --- | --- | --- | --- |
| Endpoint Measure, % achieved | Week 12  MIRI 300 mg IV N=811^a^ | Week 12  MIRI 300 mg IV N=172 | Week 28  MIRI 300 mg IV/200 mg SC N=172 |
| Clinical remission | 193 (24%) | 36 (21%) | 62 (36%) |
| Clinical response | 515 (64%) | 108 (63%) | 106 (62%) |
| Endoscopic remission | 285 (35%) | 54 (31%) | 76 (44%) |
| Endoscopic normalization | --- | 13 (7.6%) | 35 (20%) |
| Symptomatic remission | 371 (46%) | 77 (45%) | 94 (55%) |

^a^ Patients with rounded UNRS ≥3 at LUCENT-1 baseline.

Data are n (%). IV=Intravenous; MIRI=Mirikizumab; N=Number of Patients in the Analysis Population; SC=Subcutaneous. % change in clinical remission rate from Week 12 to Week 28 is 72.2% increase. % change for endoscopic normalization rate is a 169.2% improvement from Week 12 to Week 28.

Supplemental Table 3. Baseline Characteristics Between Patients Who Achieved Clinical Remission With and Without BU Remission

|  | **Clinical remission and BU remission**  **(N=36)** | **Clinical remission without BU remission (N=26)** |
| --- | --- | --- |
| **Baseline age at diagnosis** |  |  |
| <40 years | 32 (88.9) | 21 (80.8) |
| ≥40 years | 4 (11.1) | 5 (19.2) |
| **Sex** |  |  |
| Male | 20 (55.6) | 16 (61.5) |
| Female | 16 (44.4) | 10 (38.5) |
| **Baseline duration of UC** |  |  |
| <7 years | 17(47.2) | 14(53.8) |
| ≥7 years | 19(52.8) | 12(46.2) |
| **Baseline IBDQ total score** |  |  |
| ≥100 | 19(52.8) | 9(34.6) |
| <100 | 17(47.2) | 16(61.5) |
| **Baseline Urgency NRS** |  |  |
| ≤6 | 11 (30.6) | 5 (19.2) |
| >6 | 25 (69.4) | 21 (80.8) |
| **Baseline abdominal pain NRS** |  |  |
| ≤4 | 7 (19.4) | 5 (19.2) |
| >4 | 29 (80.6) | 21 (80.8) |
| **Baseline MMS** |  |  |
| Moderate (4-6) | 17 (47.2) | 8 (30.8) |
| Severe (7-9) | 19 (52.8) | 18 (69.2) |
| **Disease location** |  |  |
| Extensive UC/pancolitis | 14 (38.9) | 12 (46.2) |
| Others (left-sided colitis, proctitis) | 22 (61.1) | 14 (53.8) |
| **Loose stool** |  |  |
| Baseline Yes (6-7) | 31 (86.1) | 23 (88.5) |
| Baseline No (1-5) | 5 (13.9) | 3 (11.5) |
| Week 12 Yes (6-7) | 12 (33.3) | 5 (19.2) |
| Week 12 No (1-5) | 24 (66.7) | 21 (80.8) |
| Week 28 Yes (6-7) | 8 (22.2) | 10 (38.5) |
| Week 28 No (1-5) | 28 (77.8) | 16 (61.5) |
| **Prior biologic** |  |  |
| Failed | 11 (30.6) | 6 (23.1) |
| Not failed | 25 (69.4) | 20 (76.9) |

Data presented are n (%).

Abbreviations: BU = Bowel Urgency; IBDQ = Inflammatory Bowel Disease Questionnaire; MMS = Modified Mayo Score; NRS = Numeric Rating Scale; N = Number of Patients in the Analysis Set; N = Number of Patients in the Specified Category; UC = Ulcerative Colitis

Supplemental Table 4. Proportion Of Patients Achieving the Composite Endpoint of ES = 1 or ES = 0 With BU Remission at Week 28

|  | **Week 28**  **n/N (%)** |
| --- | --- |
| **BU remission and ES = 0 among participants with ES = 0** | 16/35 (45.7%) |
| **BU remission and ES = 1 among participants with ES = 1** | 23/41 (56.1%) |

BU Remission was defined as UNRS ≤1. BU=Bowel Urgency; ES= Endoscopic Subscore; N = number of patients in the analysis set; UNRS = Urgency Numeric Rating Scale.

Supplemental Figure 1. LUCENT-URGE Study Design. ​LUCENT-URGE was a multicenter, Phase 3b, open-label, single-arm study. Dashed arrow indicates the participant moves directly from Visit 10 to the Continued Access Period. ^a^The screening endoscopy must occur within 14 days prior to Visit 2. ^b^Participants who are eligible for continued access should move directly from Visit 10 to Visit 501, on the same day, if possible. Visit 801 should not be performed. CAP=Continued Access Period; E=Endoscopy; IV=Intravenous; LDV=Last Dosing Visit; LV=Last Visit; MIRI=Mirikizumab; Q4W=Every 4 Weeks; SC=Subcutaneous; V=Visit; W=Week.


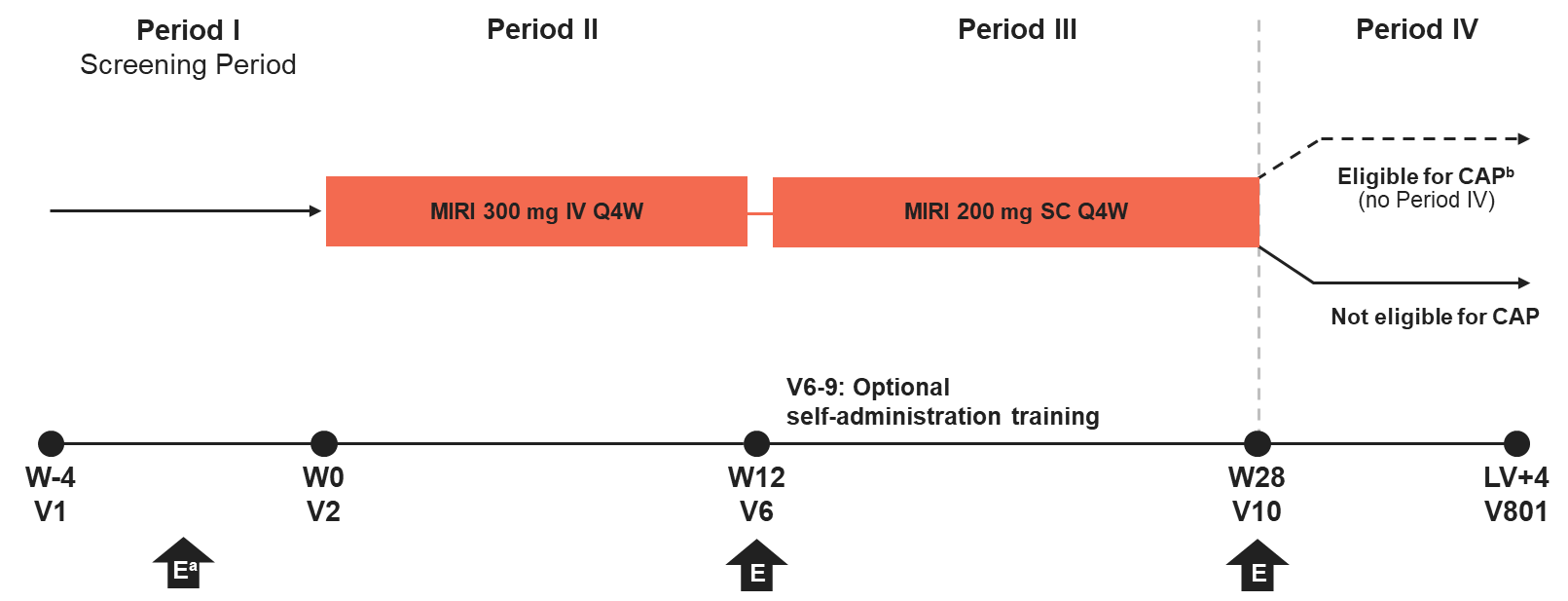


**
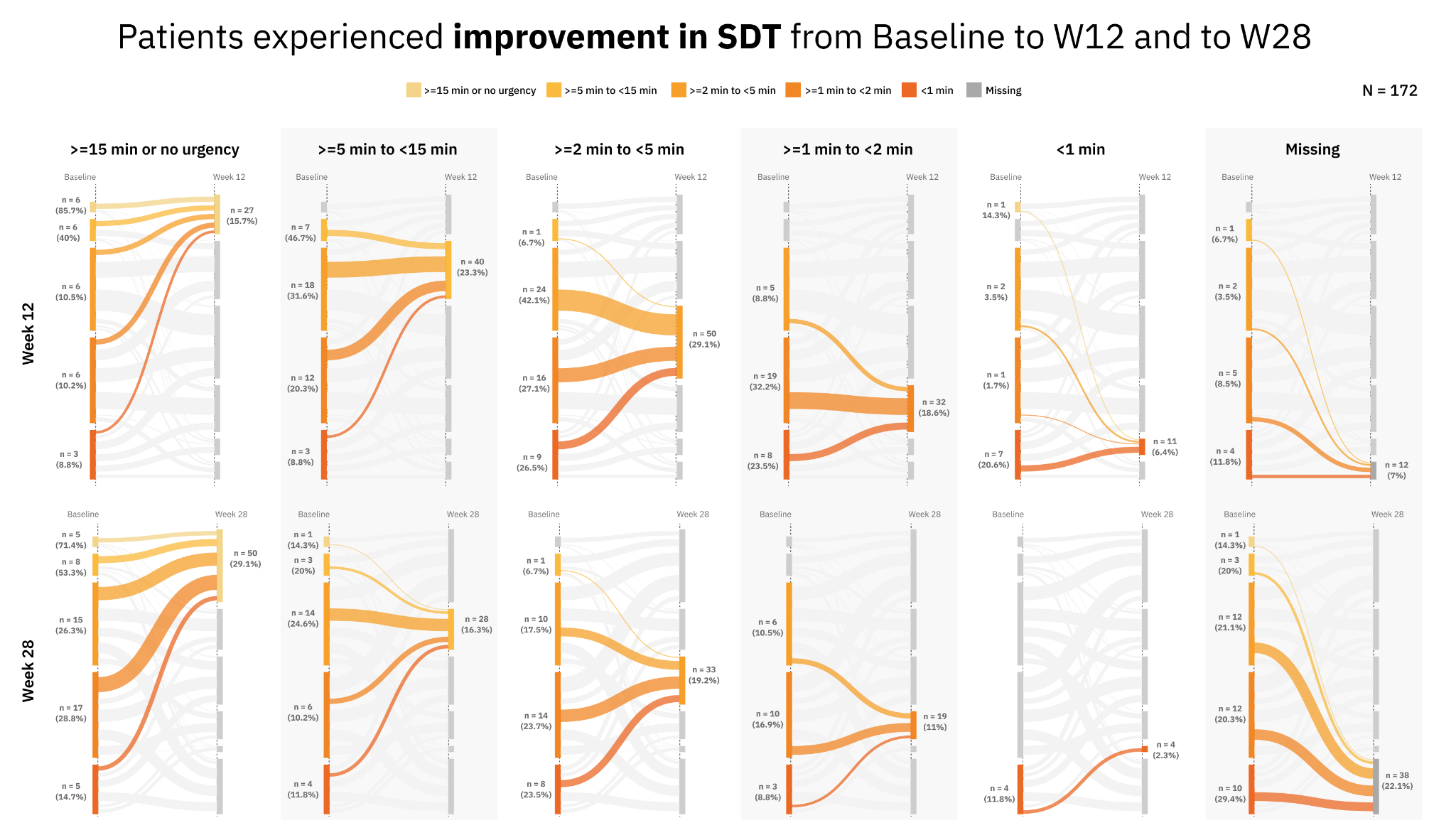
Supplemental Figure 2. Patients experienced improvement in Stool Deferral Time from Baseline to W12 and to W28**

Supplemental Figure 3. UNRS Clinically Meaning Improvement, NRI. UNRS score is collected in the patient's daily diary and calculated weekly by averaging all available UNRS responses for the 7-day week. If 4 or more UNRS responses are missing, the patient's UNRS score will be considered as missing for the week. Patients are considered nonresponders if they do not meet the categorical efficacy criteria or have missing clinical efficacy data at a time point of interest. UNRS CMI is defined as change from baseline in the UNRS Score is <= -3 (i.e., decrease from baseline >=3). CMI = clinically meaningful improvement; Miri = mirikizumab; N = number of patients in the analysis set; NRI = nonresponder imputation; UNRS = Urgency Numeric Rating Scale.


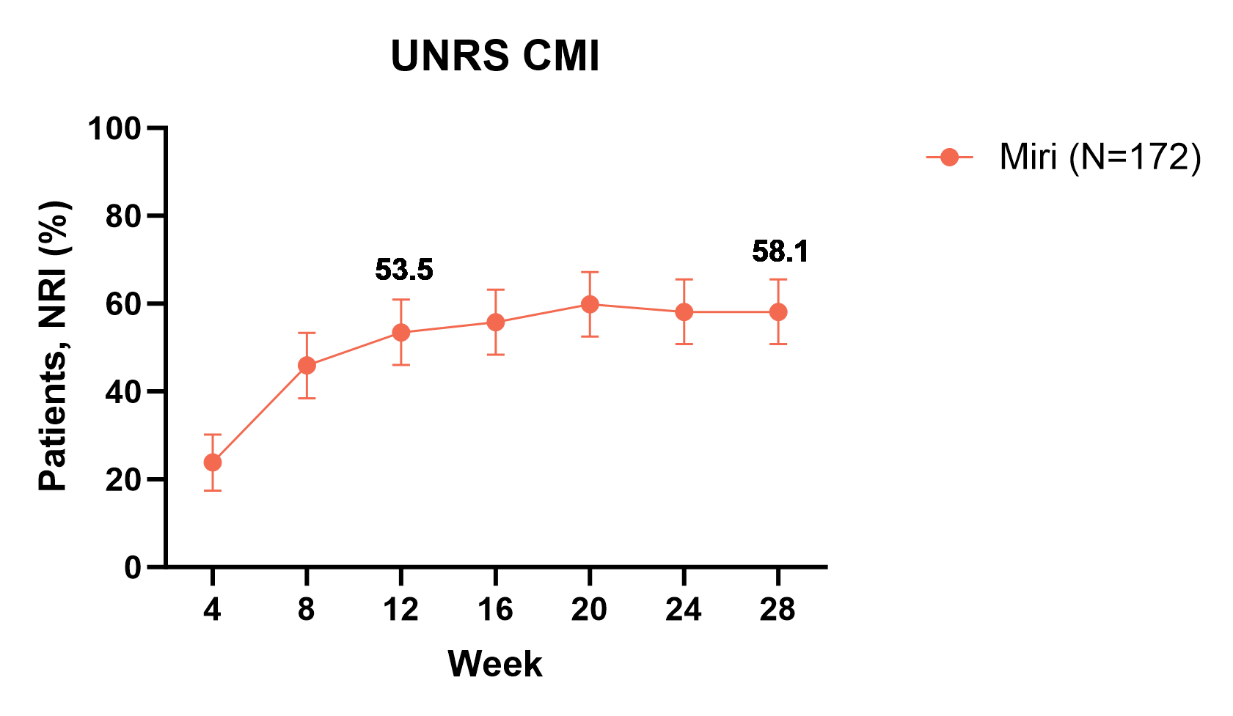

Supplement: jjag049_Supplementary_Data [file jjag049_supplementary_data.zip › Supplemental Materials_clean_13March26.docx]
